# Classic Hodgkin Lymphoma Beyond the Lymph Node: A Systemic Immunobiological Paradigm

**DOI:** 10.3390/cancers18111813

**Published:** 2026-06-01

**Authors:** Antonino Carbone, Annunziata Gloghini

**Affiliations:** 1 Centro di Riferimento Oncologico, Istituto di Ricovero e Cura a Carattere Scientifico, National Cancer Institute, 33081 Aviano, Italy; 2 Department of Advanced Diagnostics, Fondazione IRCCS, Istituto Tumori Milano, 20133 Milano, Italy; annunziata.gloghini@istitutotumori.mi.it

**Keywords:** classic Hodgkin lymphoma, systemic disease, immunobiological disorder, HRS cells, ctDNA, peripheral immune signatures, T-cell exhaustion, systemic markers, integrating monitoring

## Abstract

Classic Hodgkin lymphoma (cHL) has traditionally been conceptualized as a malignancy confined to lymphoid tissues, with disease extent defined primarily by anatomical staging systems. While this framework has guided clinical management for decades, it incompletely captures the biological complexity of cHL. Emerging evidence from molecular, immunological, and translational studies supports a reinterpretation of cHL as a systemic immunobiological disease rather than a purely nodal malignancy. A defining feature of cHL is the rarity of malignant Hodgkin and Reed–Sternberg (HRS) cells, which orchestrate a highly structured tumor microenvironment through constitutive activation of signaling pathways, including NF-κB and JAK/STAT, and through expression of immune checkpoint ligands. Beyond local effects, HRS cells secrete cytokines, chemokines, and extracellular vesicles that enter the systemic circulation, promoting widespread immune reprogramming. This includes T-cell exhaustion, expansion of regulatory T cells, and activation of immunosuppressive myeloid populations, which collectively shape host immunity beyond the lymph node. Circulating tumor DNA (ctDNA) and soluble mediators such as thymus and activation-regulated chemokine (TARC/CCL17) provide measurable evidence of systemic disease activity and enable dynamic monitoring of tumor burden. These biological insights help explain key clinical features of cHL, including constitutional (“B”) symptoms, extranodal involvement, and heterogeneous patterns of treatment response and resistance. Importantly, integration of ctDNA kinetics, peripheral immune profiling, and functional imaging offers a multidimensional framework for disease assessment that overcomes the limitations of conventional staging systems. Therapeutically, the efficacy of immune checkpoint inhibitors underscores the central role of systemic immune dysregulation, while emerging biomarker-driven strategies support adaptive and personalized approaches to treatment. Collectively, these findings support a paradigm shift toward understanding cHL as a systemic immunobiological disease. This framework has important implications for disease monitoring, therapeutic decision-making, and future research, paving the way for biology-driven, precision medicine approaches in cHL.

## Simple Summary

Classic Hodgkin lymphoma (cHL) is increasingly recognized as a systemic immunobiological disease rather than a malignancy confined to lymphoid sites. Although Hodgkin Reed–Sternberg (HRS) cells are rare, they profoundly shape the tumor microenvironment through constitutive NF-κB and JAK/STAT signaling and expression of immune checkpoint ligands. HRS-derived cytokines, chemokines, and extracellular vesicles disseminate into the bloodstream, driving systemic immune alterations such as T-cell exhaustion, regulatory T-cell expansion, and activation of immunosuppressive myeloid populations. Circulating biomarkers, including ctDNA and TARC/CCL17, reflect systemic disease activity and enable real-time monitoring. These biological insights explain clinical features such as B symptoms, extranodal spread, and variable treatment responses. Integrating ctDNA kinetics, peripheral immune profiling, and functional imaging provides a multidimensional assessment that surpasses conventional anatomic staging. The success of immune checkpoint blockade highlights the centrality of immune dysregulation, supporting a shift toward biomarker-driven, precision-medicine approaches in cHL.

## 1. Introduction

Classic Hodgkin lymphoma (cHL) has long been conceptualized as a malignancy of the lymphatic system, characterized by orderly, contiguous spread across nodal regions [1,2,3]. This anatomical framework, formalized in staging systems such as Ann Arbor and Lugano, has been central to clinical management and has contributed to the high curability of cHL [4,5]. However, this paradigm provides an incomplete representation of disease biology and does not fully account for several key clinical and molecular features that define cHL.

A distinctive hallmark of cHL is the rarity of malignant Hodgkin Reed–Sternberg (HRS) cells, which typically constitute less than 1–10% of tumor cellularity [6]. The tumor bulk is instead composed of a complex and highly structured microenvironment, including T cells, macrophages, and stromal elements, actively recruited and reprogrammed by HRS cells [2,7]. This architecture indicates that cHL is not primarily driven by tumor mass but by dynamic tumor–host interactions. Constitutive activation of signaling pathways such as Nuclear Factor kappa-light-chain-enhancer of activated B cells (NF-κB) and Janus Kinase/Signal Transducer and Activator of Transcription (JAK/STAT), along with genetic alterations including 9p24.1 amplification, enables HRS cells to evade immune surveillance and orchestrate an immunosuppressive ecosystem [2,7,8].

Importantly, accumulating evidence indicates that these interactions extend beyond the lymph node. Circulating tumor DNA (ctDNA) [9,10], soluble mediators such as thymus and activation-regulated chemokine (TARC/CCL17) [11], and systemic alterations in peripheral immune compartments provide converging evidence that cHL exerts effects at a whole-organism level [12]. Clinically, this systemic dimension is reflected by constitutional (“B”) symptoms, extranodal involvement, and heterogeneous patterns of treatment response that are not fully explained by anatomical disease burden alone [13].

These observations challenge the traditional nodal-centric view of cHL and support a paradigm shift toward understanding the disease as a systemic immunobiological entity. In this framework, HRS cells function as central regulators of immune dynamics, capable of inducing coordinated changes across multiple anatomical compartments through cytokine signaling, immune checkpoint activation, and modulation of both adaptive and innate immunity [14,15].

In this review, we integrate biological, translational, and clinical evidence supporting this reinterpretation of cHL. We discuss the role of systemic immune reprogramming, the contribution of circulating biomarkers such as ctDNA, and the implications of spatial and temporal heterogeneity. Finally, we explore how this paradigm informs disease assessment, therapeutic strategies, and future research directions, highlighting the transition from anatomy-based models toward dynamic, biology-driven approaches.

Relevant literature was identified through PubMed and Web of Science searches (last updated January 2026) using terms related to ‘Hodgkin lymphoma’, ‘tumor microenvironment’, ‘immune evasion’, ‘ctDNA’, and ‘immune profiling’. We prioritized peer-reviewed original studies and high-quality reviews focusing on biological and systemic aspects of cHL. Additionally, this review integrates knowledge and findings from the authors’ own research experience, supported by extensive engagement with current oncology literature.

## 2. Classic HL as a Systemic Immunobiological Disorder

### 2.1. The Central Role of HRS Cells Beyond Rarity

HRS cells represent a unique biological paradox in cHL, accounting for less than 1–10% of tumor cellularity yet exerting dominant control over disease biology [6]. Their pathogenic relevance derives from constitutive activation of key signaling pathways, most notably NF-κB and JAK/STAT, which are essential for HRS cell survival and proliferation [2]. These pathways are frequently driven by genetic lesions and microenvironmental stimuli, leading to sustained transcription of anti-apoptotic and immunomodulatory programs [16]. A defining feature of HRS cells is their ability to orchestrate immune evasion through overexpression of immune checkpoint ligands, particularly PD-L1 and PD-L2, often driven by 9p24.1 alterations and JAK/STAT signaling [17,18]. Noteworthy, the 9p24.1 amplicon includes JAK2 in addition to PD-L1 and PD-L2, and JAK2 overexpression further augments PD-L1 transcription through constitutive activation of JAK/STAT signaling. Furthermore, loss-of-function mutations in SOCS1, a negative regulator of JAK/STAT signaling, further potentiate STAT hyperactivation in a substantial subset of cHL cases, reinforcing PD-L1 upregulation and immune evasion. PD-1 is highly expressed on tumor-infiltrating CD4+ and CD8+ T cells in cHL, reflecting chronic antigenic stimulation and functional exhaustion. This sets the basis for the profound clinical activity of PD-1 blockade in this disease. The engagement of the PD-1 axis results in functional inhibition of T cells, promoting immune escape and contributing to the characteristic immunosuppressive microenvironment of cHL [18]. Furthermore, EBV-positive cHL, particularly in mixed cellularity cases, displays expression of LMP1, which constitutively activates NF-κB and JAK/STAT signaling. This viral program contributes to immune evasion, cytokine production, and enhanced PD-L1 expression (Table 1).

HRS cells actively remodel their environment through secretion of cytokines and chemokines that recruit CD4+ T cells, regulatory T cells, and macrophages [2,7,19,20]. These recruited cells are subsequently polarized toward phenotypes that support tumor persistence. Importantly, these soluble mediators enter systemic circulation, indicating that the influence of HRS cells extends beyond the local microenvironment [13].

In addition to lymphoid and myeloid subsets, eosinophils and mast cells are frequently enriched in the cHL microenvironment. These populations are recruited through cytokines such as IL-5 and CCL5 and contribute to tissue remodeling, angiogenesis, and support of HRS cell survival. HRS cells secrete a broad array of chemokines, including TARC/CCL17 and CCL22 (attracting CCR4+ Th2 and Treg cells), CCL5 (recruiting eosinophils and mast cells), and cytokines such as IL-5 and IL-13, which orchestrate the characteristic Th2-skewed and immunosuppressive TME.

Therefore, the capacity to modulate immune responses beyond local effects positions HRS cells as central regulators of host immunity rather than merely rare malignant elements, supporting a systemic model of cHL pathogenesis [16].

### 2.2. Systemic Immune Reprogramming: Effects on Peripheral Immune Compartments

**Figure 1:**
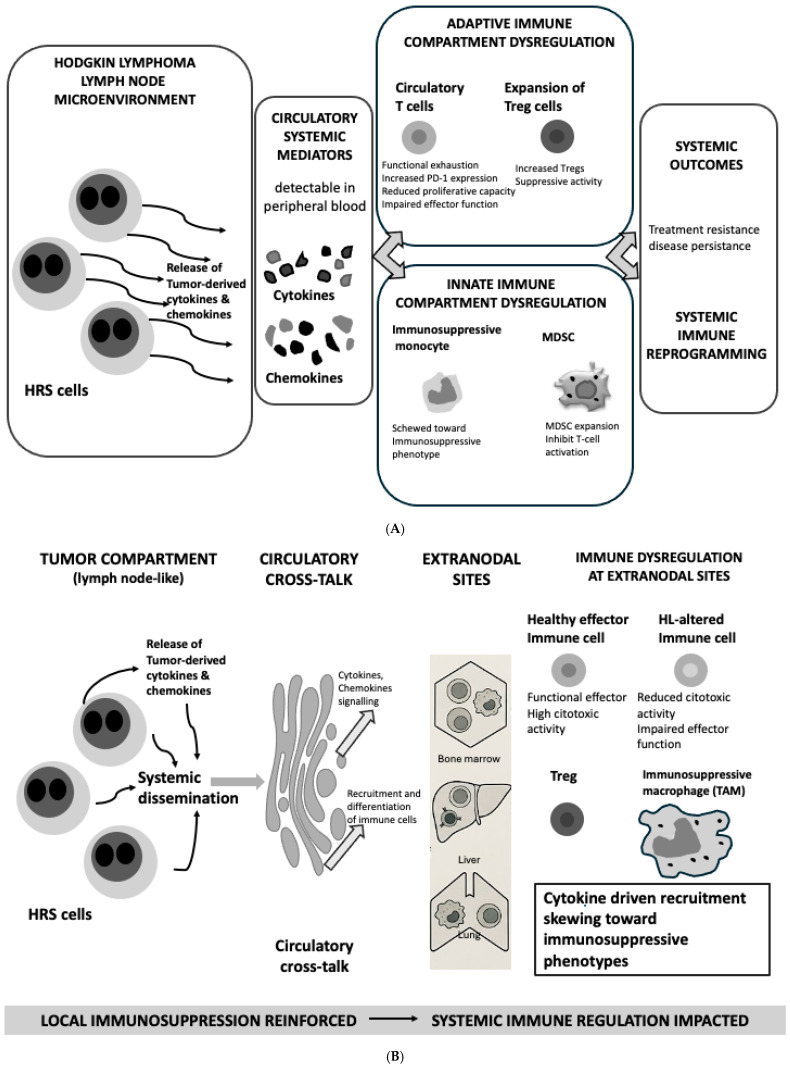
(**A**) **Systemic impact of classic Hodgkin lymphoma on peripheral immune compartments.** Schematic illustration of systemic immune dysregulation in classic Hodgkin lymphoma (cHL) extending beyond the lymph node microenvironment into the peripheral blood. Classic HL is associated with coordinated alterations in both adaptive and innate immune compartments. In the adaptive compartment, circulating T cells display features of functional exhaustion, including increased expression of inhibitory receptors such as PD-1, reduced proliferative capacity, and impaired effector function, alongside expansion of regulatory T-cells (Tregs) with suppressive activity. In the innate compartment, monocyte populations are skewed toward immunosuppressive phenotypes, with expansion of cells consistent with myeloid-derived suppressor cells (MDSCs) that inhibit T-cell activation and contribute to an immunoregulatory milieu. These alterations are driven, at least in part, by tumor-derived cytokines and chemokines released by Hodgkin Reed–Sternberg (HRS) cells and detectable in the circulation. Collectively, these changes reflect systemic immune reprogramming that may contribute to treatment resistance and disease persistence, while remaining dynamic and potentially reversible with effective therapy. (**B**) **Immune dysfunction at extranodal sites in classic Hodgkin lymphoma**. Schematic representation of immune dysregulation at extranodal sites in classic Hodgkin lymphoma (cHL). Tumor-derived factors produced by Hodgkin Reed–Sternberg (HRS) cells, including cytokines and chemokines, enter the systemic circulation and influence immune responses in distant organs such as bone marrow, liver, and lung. At these sites, immune cell populations exhibit functional alterations characterized by reduced cytotoxic activity, impaired effector function, and skewing toward immunosuppressive phenotypes, including regulatory T-cells (Tregs) and macrophage populations. Chemokine-driven recruitment and differentiation of immune cells contribute to the accumulation of functionally altered populations, reinforcing local immunosuppression. Involvement of hematopoietic compartments such as the bone marrow may further impact systemic immune regulation. This model illustrates how tumor–host interactions extend beyond the lymph node, supporting a systemic framework of cHL in which coordinated immune alterations occur across multiple anatomical compartments. TAM, Tumor-associated macrophage.

Classic HL is associated with widespread immune perturbations affecting both adaptive and innate compartments [21]. Elevated circulating cytokines, including TARC/CCL17, IL-6, and IL-10, correlate with disease activity and contribute to systemic inflammation [11,22,23]. These mediators influence immune cell trafficking and differentiation, promoting expansion of immunosuppressive populations (Table 2, Figure 1A).

Classic HL is characterized not only by a highly structured tumor microenvironment but also by systemic perturbations of peripheral immunity that mirror and extend local immune dysregulation [12,16]. Peripheral blood analyses in patients with active cHL consistently reveal quantitative and functional alterations affecting both adaptive and innate immune compartments, supporting a model of disease-driven, body-wide immune reprogramming [24].

Circulating T cells display hallmarks of functional exhaustion [14], including upregulation of inhibitory receptors such as PD-1 and impaired effector responses [25]. Notably, this phenotype is not confined to tumor-infiltrating lymphocytes [26,27] but is also detectable in peripheral blood, indicating that immune checkpoint–mediated dysfunction operates at a systemic level [25]. In parallel, expansion of regulatory T cells contributes to a suppressive immune tone, potentially limiting effective anti-tumor immunity [28] (Table 2).

The myeloid compartment is similarly altered. Increased frequencies of circulating monocytes with immunosuppressive features, along with signatures consistent with myeloid-derived suppressor cells (MDSCs), have been associated with adverse outcomes and may reflect systemic propagation of tumor-induced immune regulation [29]. These alterations are likely driven, at least in part, by soluble mediators produced by HRS cells, including TARC/CCL17 and other cytokines, which are detectable in the circulation and correlate with disease burden [11,22] (Table 2).

Importantly, these systemic immune abnormalities are dynamic. Restoration of immune competence following effective therapy, particularly with immune checkpoint blockade, underscores their functional relevance and reversibility [30,31]. However, incomplete normalization in some patients suggests that persistent immune dysregulation may contribute to treatment resistance and relapse.

### 2.3. Crosstalk Beyond the Lymph Node

Emerging evidence supports the concept that cHL is associated with conditioning of immune microenvironments beyond the primary tumor site, mediated by circulating cytokines and chemokines, including TARC/CCL17, which are detectable in peripheral blood and correlate with disease burden and treatment response [11,22]. These soluble mediators are not confined to the tumor microenvironment but act systemically, influencing immune cell trafficking and functional polarization in distant tissues. Mechanistically, CCL17 promotes recruitment of CCR4-positive T-helper 2 (Th2) and regulatory T cells, contributing to the establishment of immunosuppressive niches outside lymph nodes [23]. Tumor-driven immune dysregulation extends to extranodal compartments, where it recapitulates key features of the nodal microenvironment within a systemic framework.

Extranodal sites such as bone marrow, liver, and lung are the most frequent locations of disease involvement [15]. They are not merely passive locations of disease involvement but show diagnostic HRS cells within the proper background [32] and TME alterations [33,34]. They may represent biologically active niches shaped by tumor-derived signals and host immune responses (Figure 1B). The coordinated remodeling of the immune microenvironment supports tumor survival and may facilitate dissemination or persistence in anatomically distinct compartments. Involvement of the bone marrow further highlights the systemic nature of cHL-associated immune dysfunction [33,34]. Alterations in hematopoietic and immune regulatory processes at this site may contribute to impaired immune surveillance and sustained immunosuppression. In this model, extranodal immune dysfunction is driven by systemic tumor–host crosstalk, reinforcing the concept of cHL as a disease characterized by coordinated immune modulation across multiple anatomical compartments with direct implications for therapeutic targeting.

## 3. Biological Evidence of Disease Beyond the Lymph Node

### 3.1. Circulating Tumor DNA and Liquid Biopsy

ctDNA has emerged as a key tool for understanding cHL as a systemic disease. ctDNA consists of fragmented tumor-derived DNA released into the bloodstream and provides a minimally invasive means to capture tumor genetics and disease dynamics. Recent studies demonstrate that ctDNA is readily detectable in classical cHL, reflecting the high turnover of HRS cells and enabling comprehensive molecular profiling from plasma [10,35] (Table 3). Importantly, ctDNA levels correlate with tumor burden and treatment response, supporting its role as a dynamic biomarker of disease activity [36]. Longitudinal analyses have shown that ctDNA kinetics can track therapeutic response and may identify molecular persistence or relapse earlier than conventional imaging in selected settings [37]. Moreover, ctDNA integrates signals from multiple disease sites, overcoming the spatial limitations of single-site biopsy and capturing inter- and intra-lesional heterogeneity [38].

In cHL, ctDNA profiling has revealed concordance with tumor tissue genomics while also uncovering additional variants, highlighting its potential to reflect systemic disease architecture [9,39,40]. Advances in next-generation sequencing and digital PCR have further improved sensitivity, enabling applications in minimal residual disease (MRD) detection and risk stratification [41].

Although standardization and clinical validation remain ongoing, liquid biopsy approaches based on ctDNA are poised to complement imaging and redefine disease monitoring. ctDNA has emerged as a promising biomarker for MRD assessment and early relapse detection. Importantly, ctDNA integrates signals across multiple tumor sites, addressing limitations of single-site biopsy. Longitudinal monitoring enables assessment of disease kinetics and clonal evolution, offering insights into mechanisms of treatment resistance [10]. Collectively, these findings position ctDNA as a central component of biology-driven assessment in cHL, supporting its reinterpretation as a systemically distributed malignancy.

The clinical utility of ctDNA in cHL is closely linked to its sensitivity for detection, its dynamic behavior over time, and its potential role in disease monitoring. Advances in next-generation sequencing (NGS) technologies have significantly improved the sensitivity of ctDNA detection, enabling the identification of low-frequency tumor-derived variants in plasma and allowing for minimally invasive molecular profiling [42].

ctDNA kinetics provide critical insight into disease dynamics. Early changes in ctDNA levels during therapy have been shown to correlate with treatment response, with rapid clearance associated with favorable outcomes, whereas persistent or re-emerging ctDNA is strongly associated with residual disease and subsequent relapse [43]. Early studies suggest that ctDNA may provide complementary or potentially more sensitive early response information compared with interim PET, although systematic validation is ongoing. From a clinical perspective, ctDNA offers the potential to complement or even refine conventional imaging approaches. Unlike positron emission tomography/computed tomography (PET/CT), which reflects metabolic activity, ctDNA provides a direct molecular readout of tumor burden and can detect MRD below the threshold of imaging sensitivity [41]. This capability may enable earlier therapeutic intervention and facilitate risk-adapted strategies.

Although challenges remain regarding assay standardization and optimal thresholds, ctDNA-based monitoring represents a promising step toward real-time, biology-driven assessment of cHL, with potential applications in response-adapted therapy and clinical trial design.

### 3.2. Peripheral Immune Signatures

**Figure 2:**
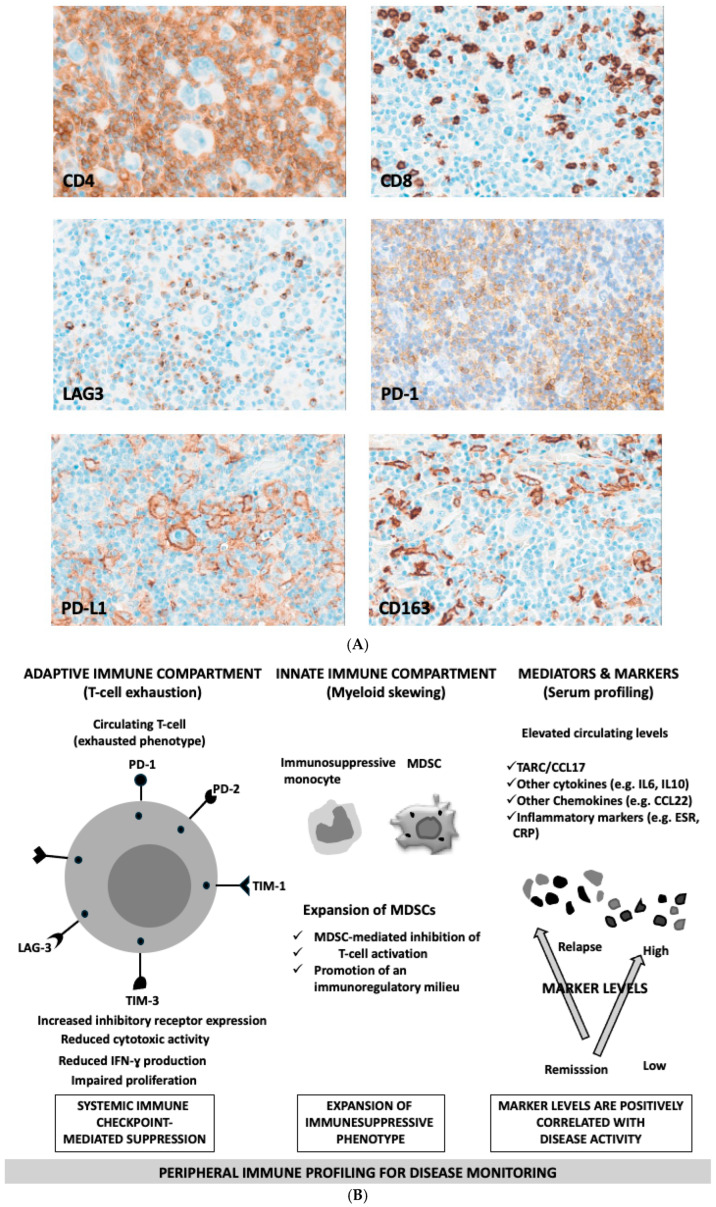
(**A**) **Spatial organization of exhausted T cells and tumor-associated macrophages in the classic Hodgkin lymphoma (cHL) tumor microenvironment**. Representative immunohistochemical staining of a cHL lymph node illustrates the cellular composition and spatial architecture of the tumor niche. Panels show T-cell subsets (CD4, CD8) together with the expression of inhibitory receptors associated with T-cell exhaustion, including PD-1 and LAG-3, as well as PD-L1 expression in the surrounding microenvironment. Tumor-associated macrophages (TAMs) indicating an immunosuppressive M2-like phenotype are identified by CD163 staining. Tumor-infiltrating lymphocytes display features of functional exhaustion through checkpoint receptor expression, while TAMs contribute to the establishment of an immunosuppressive niche. The close spatial distribution of PD-1+ T cells, PD-L1–expressing cells, and macrophages around Hodgkin Reed–Sternberg cells supports the formation of PD-1/PD-L1-enriched microenvironments that promote immune evasion. Original magnification 40X. (**B**) **Peripheral immune signatures in classic Hodgkin lymphoma: T-cell exhaustion, myeloid skewing, and systemic markers. Schematic representation of systemic immune dysregulation in classic** Hodgkin lymphoma (cHL) as reflected by peripheral immune signatures. Circulating T cells display features of functional exhaustion, including increased expression of inhibitory receptors such as PD-1, reduced cytotoxic activity, and impaired effector function, consistent with systemic immune checkpoint–mediated suppression. In parallel, the myeloid compartment is skewed toward immunosuppressive phenotypes, with expansion of cells consistent with myeloid-derived suppressor cells (MDSCs) that inhibit T-cell activation and promote an immunoregulatory milieu. Elevated circulating levels of cytokines and chemokines, including thymus and activation-regulated chemokine (TARC/CCL17), together with inflammatory markers, reflect systemic immune activation and correlate with disease activity. Collectively, these alterations illustrate coordinated dysfunction across adaptive and innate immune compartments, supporting a systemic model of cHL and highlighting the potential role of peripheral immune profiling in disease monitoring and therapeutic stratification. ESR, erythrocyte sedimentation rate; CRP, C-reactive protein.

Peripheral immune signatures in cHL reflect a coordinated state of systemic immune dysregulation involving both adaptive and innate compartments. Among the most consistently reported features is T-cell dysfunction characterized by an exhausted phenotype [15]. Circulating T cells frequently exhibit increased expression of inhibitory receptors, including PD-1, along with reduced proliferative capacity and impaired effector function [14,28]. This phenotype parallels observations within the tumor microenvironment and indicates that immune checkpoint–mediated suppression extends beyond localized disease sites [12,20] (Table 3, Figure 2A,B).

In addition to T-cell alterations, changes in the myeloid compartment contribute to systemic immune modulation. Peripheral blood analyses have demonstrated shifts in monocyte populations toward phenotypes associated with immunosuppressive activity. Cells with features consistent with MDSCs have been identified in cHL and are capable of inhibiting T-cell activation and cytokine production [29]. These myeloid populations may arise or expand under the influence of tumor-derived factors and are increasingly recognized as contributors to disease-associated immune dysfunction [19,20] (Table 3).

Systemic soluble markers further support the presence of widespread immune perturbation. Elevated circulating levels of cytokines and chemokines, including thymus and activation-regulated chemokine (TARC/CCL17), correlate with disease activity and tumor burden [12,23]. These mediators are involved in immune cell recruitment and polarization and may contribute to both local and peripheral immune alterations. In addition, inflammatory markers and acute-phase reactants reflect a broader state of systemic immune activation [44].

Importantly, these peripheral immune signatures are dynamic and may change in response to therapy. Restoration of immune competence following treatment, particularly with immune checkpoint blockade, underscores their biological relevance and reversibility [45]. However, incomplete normalization in some patients suggests that persistent immune dysregulation may contribute to treatment resistance and disease recurrence. Collectively, the convergence of T-cell exhaustion, myeloid skewing, and circulating immune markers highlights the potential role of peripheral immune profiling in disease monitoring and therapeutic stratification. Peripheral immune profiling is a promising investigational tool but currently lacks standardized assays, reference ranges, and validated clinical thresholds.

### 3.3. Temporal and Spatial Heterogeneity

Classic HL exhibits both temporal and spatial heterogeneity, reflecting dynamic interactions between tumor cells, the microenvironment, and therapeutic pressure. Although HRS cells share core biological features, emerging data indicate variability in genetic alterations and immune context over time [10,14]. Spatial heterogeneity in cHL is shaped largely by differences in the tumor microenvironment. Distinct anatomical compartments may harbor variations in immune cell composition, cytokine gradients, and stromal interactions, all of which influence tumor behavior.

Temporal heterogeneity arises from clonal evolution and selective pressures imposed by therapy. Longitudinal analyses using ctDNA have demonstrated that cHL evolves over time, with changes in mutational profiles and subclonal architecture detectable during treatment and at relapse [10,36]. These findings indicate that disease progression is not static but reflects ongoing biological adaptation. ctDNA provides a systemic readout of this heterogeneity, capturing signals from multiple sites and enabling longitudinal assessment [10]. This approach may provide a more comprehensive view of disease biology than conventional biopsy.

## 4. Clinical Correlates of a Systemic Disease

Beyond nodal involvement, cHL is frequently associated with constitutional (“B”) symptoms—including fever, night sweats, and weight loss—which are closely linked to systemic cytokine production and inflammatory signaling [6,15]. Elevated levels of circulating mediators such as interleukin-6 and TARC/CCL17 correlate with disease activity and symptom burden.

Extranodal involvement represents another key clinical correlate. Disease localization in sites such as bone marrow, liver, and lung reflects dissemination beyond lymphoid tissues and is associated with more advanced disease and distinct clinical behavior [15]. Although many patients achieve durable remission, a subset experiences relapse at sites distinct from initial presentation, suggesting the presence of residual disease not fully captured by conventional imaging [39]. In this context, biomarkers such as ctDNA and peripheral immune signatures provide additional insights into disease dynamics and may identify molecular persistence despite apparent radiologic remission (Table 4).

While frontline therapies achieve high cure rates, a subset of patients exhibits primary refractory disease or relapse, reflecting underlying biological heterogeneity and dynamic tumor–host interactions [6]. Emerging evidence suggests that treatment response in cHL is not determined solely by tumor burden, but also by the state of the immune microenvironment and systemic immune competence [20]. Immune checkpoint blockade has demonstrated substantial efficacy in relapsed or refractory cHL, underscoring the central role of PD-1/PD-L1–mediated immune evasion [46]. However, not all patients respond, and resistance mechanisms are increasingly recognized. These may include, as discussed above, persistent T-cell dysfunction, inadequate immune reactivation, and compensatory immunosuppressive pathways involving regulatory T cells or myeloid populations.

In this context, circulating biomarkers such as ctDNA provide insight into response dynamics. Response and resistance in cHL are governed by systemic biological processes involving both tumor-intrinsic and immune-mediated mechanisms. Integrating molecular and immune biomarkers into clinical assessment may therefore improve risk stratification and guide adaptive therapeutic strategies aimed at overcoming resistance.

## 5. Rethinking Disease Assessment

### 5.1. Limits of Current Staging Systems

Current staging systems in cHL, including the Ann Arbor and Lugano classifications, are fundamentally based on anatomical disease distribution and patterns of nodal and extranodal involvement [4,5]. While these frameworks have been instrumental in standardizing disease assessment and guiding therapy, they provide only a partial representation of disease biology and fail to capture the systemic and dynamic nature of cHL.

One key limitation is the reliance on imaging, particularly PET/CT, which reflects metabolic activity but does not directly measure tumor biology or molecular disease burden [39]. Although PET-adapted strategies have improved risk stratification and treatment personalization [47,48], they remain constrained by their inability to detect MRD at a molecular level. Moreover, patients with similar stage and imaging findings may exhibit markedly different biological profiles, including variations in immune microenvironment composition, cytokine levels, and ctDNA dynamics, which are increasingly recognized as determinants of clinical outcome. Another limitation lies in the static nature of current staging systems, which provide a snapshot of disease at diagnosis but do not capture temporal evolution during treatment. As discussed above, cHL is a dynamic disease influenced by therapy-induced selection pressures, and changes in disease biology over time may not be reflected by conventional staging approaches.

These limitations highlight the need for integrating biological markers into disease assessment. Emerging tools such as ctDNA analysis and peripheral immune profiling offer the potential to complement anatomical staging by providing real-time insights into disease activity and systemic biology.

### 5.2. Integrating Biomarkers with Imaging

The combined use of ctDNA and PET/CT enables a more comprehensive assessment of cHL by integrating anatomical, metabolic, and molecular information [39]. This multimodal approach may improve risk stratification, refine response-adapted strategies, and support earlier identification of high-risk patients. As evidence continues to accumulate, integration of biomarkers with imaging is likely to play an increasingly central role in biology-driven disease monitoring [43].

### 5.3. Toward Dynamic and Biology-Driven Monitoring

**Figure 3:**
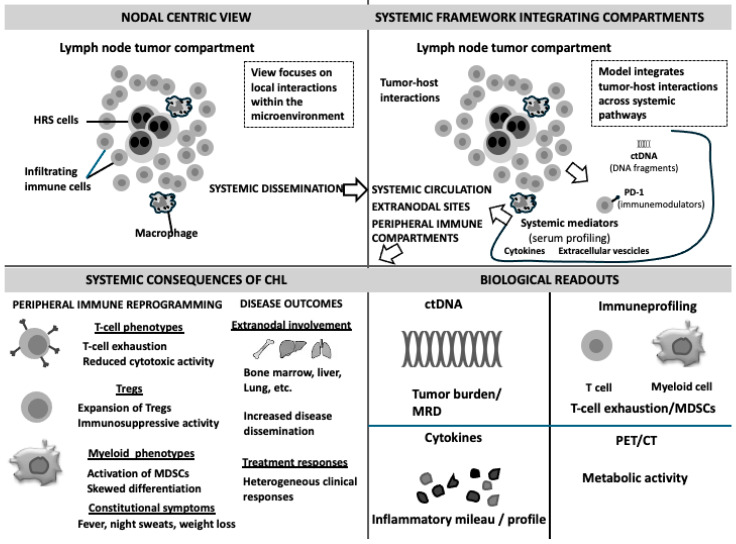
Dynamic, biology-driven monitoring framework in classic Hodgkin lymphoma. Schematic representation of integrated disease monitoring in classic Hodgkin lymphoma (cHL). Hodgkin Reed–Sternberg (HRS) cells release ctDNA, cytokines, and immune-modulatory signals into the systemic circulation. These factors enable multiparametric assessment through ctDNA analysis, peripheral immune profiling, cytokine measurement, and PET/CT imaging. Integration of molecular, immune, and functional data provides a dynamic representation of disease activity, supporting early response assessment, detection of minimal residual disease, and adaptive therapeutic strategies. This framework reflects the systemic and evolving nature of HL and highlights the transition from static, anatomy-based evaluation to biology-driven monitoring.

Traditional approaches, centered on imaging and baseline staging, provide limited insight into the underlying molecular and immunological processes that drive disease progression and therapeutic response (Table 5, Figure 3). CtDNA provides a direct molecular readout of these dynamics. As ctDNA reflects tumor cell turnover and apoptotic release of DNA fragments, its quantitative changes over time mirror treatment-induced tumor cell killing and residual disease persistence.

In parallel, peripheral immune signatures offer complementary insights into host response. Integrating molecular and immune biomarkers with imaging enables a multidimensional assessment of cHL, capturing tumor burden, biological activity, and immune context simultaneously. This approach supports adaptive therapeutic strategies, in which treatment intensity can be modulated based on early biological response.

## 6. Therapeutic Implications

Anti–PD-1 therapies such as nivolumab and pembrolizumab have demonstrated high response rates in relapsed or refractory cHL, confirming that tumor persistence is critically dependent on immune suppression rather than intrinsic resistance alone [49,50,51]. However, variability in response highlights the importance of additional mechanisms, including persistent T-cell exhaustion, regulatory T-cell expansion, and immunosuppressive myeloid populations that may limit full immune reactivation. In this context, combining checkpoint inhibitors with agents targeting the tumor microenvironment or myeloid compartment represents a rational strategy to overcome resistance [30] (Table 6).

Several practical considerations need to be addressed before multidimensional monitoring can be broadly adopted, including harmonization of ctDNA sequencing assays, definition of clinically meaningful thresholds, optimal timing of sampling relative to treatment cycles, and integration with established PET-adapted algorithms. Prospective trials incorporating standardized ctDNA collection and paired imaging will be essential to validate these approaches.

## 7. Future Directions and Research Priorities

Future research in cHL should focus on refining the concept of cHL as a systemic immunobiological disease through integration of multi-dimensional data and longitudinal monitoring approaches. A major priority is the clinical validation of ctDNA as a biomarker for MRD and treatment response. Recent studies suggest that ctDNA kinetics can provide early and sensitive detection of residual disease and may outperform conventional imaging in selected contexts [36,39,41,43,52]. Standardization of assays, definition of clinically meaningful thresholds, and incorporation into prospective clinical trials remain essential steps for clinical implementation. In parallel, integration of immune profiling with molecular monitoring may enable a more comprehensive assessment of disease biology. Longitudinal characterization of T-cell function, myeloid populations, and cytokine networks could help identify mechanisms of resistance to immunotherapy and guide combination strategies [53].

Future clinical trials should incorporate biology-driven endpoints, including ctDNA dynamics and immune biomarkers, alongside traditional imaging-based criteria. This approach may facilitate adaptive treatment strategies and improve patient stratification. Ultimately, a deeper mechanistic understanding of systemic immune reprogramming in cHL will be essential to fully realize precision medicine approaches and to develop therapies that target both tumor cells and the broader immune context in which they reside.

## 8. Conclusions

Classic HL has traditionally been viewed as a nodal malignancy; however, emerging evidence supports its reinterpretation as a systemic immunobiological disease. The cumulative biological and clinical evidence reviewed here supports a fundamental reinterpretation of cHL as a systemic immunobiological disease. This paradigm shift is grounded in the recognition that rare HRS cells exert disproportionate influence through sustained activation of immune-modulatory pathways and secretion of soluble factors that extend their impact beyond the tumor microenvironment. Systemic dissemination of cytokines, chemokines, and ctDNA provides a mechanistic basis for coordinated immune alterations across peripheral compartments. These processes are reflected in clinical features such as B symptoms, extranodal involvement, and heterogeneous treatment responses, which cannot be fully explained by anatomical staging alone. Peripheral immune signatures, including T-cell exhaustion and myeloid skewing, further reinforce the concept of disease-wide immune reprogramming.

Importantly, this systemic perspective has direct implications for disease assessment and management. Traditional staging systems, while clinically useful, are inherently limited by their reliance on static anatomical information. In contrast, integration of molecular and immune biomarkers—particularly ctDNA and peripheral immune profiling—enables dynamic monitoring of disease activity and provides a more comprehensive representation of tumor burden and host response. The combination of these approaches with functional imaging represents a critical step toward biology-driven evaluation.

Therapeutically, the success of immune checkpoint inhibitors underscores the central role of immune evasion in HL pathogenesis and provides functional validation of its systemic nature. However, variability in response highlights the need to better understand mechanisms of resistance, including persistent immune dysfunction and microenvironmental factors. Biomarker-guided strategies offer the potential to refine treatment selection, enabling adaptive approaches that optimize efficacy while minimizing toxicity.

Looking forward, integration of multi-omic technologies, longitudinal monitoring, and biology-driven clinical endpoints will be essential to fully realize precision medicine in HL. By aligning disease classification with underlying biology, this systemic framework provides a foundation for improving risk stratification, guiding therapeutic innovation, and ultimately enhancing patient outcomes. In this context, moving beyond a nodal-centric model is not merely conceptual—it represents a necessary evolution toward a more accurate and clinically actionable understanding of cHL.

## Figures and Tables

**Table 1:** Key molecular mechanisms driving systemic classic Hodgkin lymphoma biology.

Pathway	Mechanism	Systemic Effect
NF-κB	Constitutive activation in HRS cells	Cytokine production, HRS cell survival
JAK/STAT	Cytokine signaling amplification	Immune modulation
9p24.1 amplification	PD-L1/PD-L2 overexpression	Immune evasion, checkpoint sensitivity
EBV-associated signaling	LMP1-mediated NF-κB activation, PD-L1/PD-L2 overexpression	Subset-specific biology, immune evasion, checkpoint sensitivity

HRS, Hodgkin Reed-Sternberg; LMP1, latent membrane protein.

**Table 2:** Systemic immune alterations in classic Hodgkin lymphoma.

Compartment	Alteration	Functional Impact
T cells	PD-1 upregulation, exhaustion	Reduced cytotoxicity
Tregs	Expansion	Immunosuppression
Myeloid cells	MDSC-like phenotypes	T-cell inhibition
Cytokines	TARC/CCL17, IL-6, IL-10 ↑	Systemic inflammation.

MDSC, myeloid-derived suppressor cell; ↑ overproduction.

**Table 3:** Circulating biomarkers, beyond the lymph node, in classic Hodgkin lymphoma.

Biomarker	Type	Clinical Utility
ctDNA	Molecular, tumor derived	MRD, early relapse detection
TARC/CCL17	Cytokine	Disease activity
IL6	Cytokine	Inflammation
PD-L1 expression	Immune checkpoint	Predictive biomarker
Immune profiling	Cellular	Risk stratification

**Table 4:** Clinical correlates supporting classic Hodgkin lymphoma as a systemic immunobiological disease.

Clinical Feature	Biological Basis	Systemic Implication	Clinical Relevance
B symptoms (fever, weight loss, night sweats)	Elevated cytokines (e.g., IL-6, TNF-α, TARC/CCL17) produced by HRS cells and immune infiltrate	Reflect systemic inflammatory and immune activation	Associated with disease burden and adverse prognosis
Extranodal involvement (bone marrow, liver, lung)	Tumor-driven immune modulation and trafficking of immune cells to distant tissues	Indicates disease activity beyond lymph nodes	Defines advanced-stage disease and influences risk stratification
Peripheral immune alterations	T-cell exhaustion, Treg expansion, myeloid skewing	Evidence of systemic immune reprogramming	Potential biomarkers for disease monitoring and therapeutic response
Circulating biomarkers (ctDNA, cytokines)	Release of tumor-derived DNA and soluble mediators into circulation	Captures global disease burden and dynamics	Enables MRD detection and early relapse identification
Heterogeneous treatment response	Variability in tumor biology and immune competence	Reflects systemic disease complexity	Guides need for risk-adapted and personalized therapy
Patterns of relapse (including new sites)	Residual systemic disease and clonal evolution	Suggests incomplete eradication of disease across compartments	Supports integration of molecular monitoring strategies

ctDNA, circulating tumor DNA; MRD, minimal residual disease.

**Table 5:** Toward dynamic and biology-driven monitoring in classic Hodgkin lymphoma.

Domain	Biological Basis	Monitoring Tool	Clinical Implication
Tumor burden (molecular)	Tumor DNA release from apoptotic/necrotic HRS cells	ctDNA (NGS, digital PCR)	Early response assessment, MRD detection, relapse prediction
Tumor kinetics	Dynamic changes in tumor cell turnover during therapy	Serial ctDNA measurements	Identification of responders vs. non-responders early in treatment
Immune status (adaptive)	T-cell exhaustion, PD-1 signaling, TCR repertoire changes	Peripheral immune profiling (flow cytometry, sequencing)	Prediction of response to immunotherapy
Immune status (innate)	Myeloid cell expansion, cytokine-driven immunosuppression	Cytokine panels, myeloid profiling	Identification of resistance mechanisms
Functional tumor activity	Glucose metabolism and inflammatory activity	PET/CT imaging	Standard response evaluation, anatomical context
Integrated biology	Tumor–host interaction across compartments	ctDNA + immune biomarkers + PET	Biology-driven risk stratification and adaptive therapy

HRS, Hodgkin Reed-Sternberg; ctDNA, circulating tumor DNA; MRD, minimal residual disease.

**Table 6:** Clinical therapeutic implications of the systemic model.

Strategy	Rationale	Example
Staging	Traditional: anatomical approach	Systemic approach: biological + anatomical
Checkpoint inhibition	Reverse T-cell exhaustion	Nivolumab, pembrolizumab
Biomarker-guided therapy	Adapt treatment intensity	ctDNA-guided approaches
Combination therapy	Target multiple pathways	Chemo + immunotherapy
Monitoring	Traditional: PET/CT	Systemic approach: PET/CT + ctDNA

## Data Availability

No new data were created or analyzed in this study.
